# Symmetrical peripheral gangrene caused by urosepsis: Case reports and literature review

**DOI:** 10.1097/MD.0000000000039508

**Published:** 2024-10-04

**Authors:** Yuanyuan Chen, Kai Liu, Xiujuan Xu, Gaofei Wu, Lianghua Zhu, Junjing Zha, Chuji Cheng

**Affiliations:** a Department of Critical Care Medicine, Anqing Municipal Hospital, Anqing City, Anhui Provinces, China; b Department of Cardiovascular, Anqing Municipal Hospital, Anqing City, Anhui Provinces, China.

**Keywords:** amputation, case report, DIC, sepsis, symmetrical peripheral gangrene

## Abstract

**Rationale:**

Symmetrical peripheral gangrene (SPG) is a serious and rare complication in patients with urosepsis, characterized by distal limb symmetry impairment.

**Patient concerns:**

In this study, 3 cases of SPG caused by urosepsis were reported, and the Chinese and English literature on SPG caused by urosepsis was reviewed. The demographic, clinicopathological, treatment, and follow-up data of the patients were summarized and analyzed.

**Diagnosis:**

SPG was diagnosed with clinical symptoms.

**Interventions:**

We conducted urological invasive surgery, administered anti-infective therapy, implemented fluid resuscitation and blood product transfusion, provided mechanical ventilation support, optimized myocardial contractility, administered heparin and B vitamins, utilized papaverine for vasodilation, performed hemodialysis and plasma exchange, peripheral skin warming along with other treatment modalities.

**Outcomes:**

Two patients died and 1 patient underwent autoamputation.

**Lessons:**

Our cases and literature review demonstrate that timely and accurate diagnosis, effective infection control, correction of hypoperfusion, organ function support, early management of disseminated intravascular coagulation, avoidance of premature amputation, and multidisciplinary comprehensive treatment are crucial for the successful treatment of SPG caused by urosepsis.

## 1. Introduction

Patients with urogenic sepsis account for 20% to 30% of all sepsis cases,^[1]^ and the fatality rate is 25% to 60%.^[2]^ Some of these patients die from complications rather than the infection itself. Symmetrical peripheral gangrene (SPG) is a serious and rare complication in patients with urosepsis, characterized by distal limb symmetry impairment.^[3]^ Therefore, raising awareness of SPG caused by urosepsis has important clinical significance.

## 2. Case presentation

### 2.1. Case 1

A 53-year-old female was admitted to the intensive care unit with a 3-day history of flank pain and a 1-day history of fever. She had a history of urinary calculi. The initial diagnosis was urosepsis and septic shock. After admission, we obtained the blood, urine and sputum cultures, therapeutically, initial empiric antimicrobial drugs (imipenem and cilastatin sodium), hydrocortisone, invasive ventilation, fluid resuscitation, vasopressor drugs (norepinephrine and metaraminol were titrated to a maximum of 2 µg/kg/min, 10 µg/kg/min) and CRRT were performed. And the urologists performed bilateral renal percutaneous puncture and drainage under color Doppler ultrasound positioning to extract purulent urine. On day 3, the vasopressor drugs were gradually reduced, but large purple plaques began to appear at the ends of the upper and lower limbs. Some of the examination results are shown in Tables 1 and 2. The blood culture results indicated *Klebsiella pneumoniae*. We added heparin, papaverine, and peripheral area warming. In the following 4 days, there was no obvious progress in the purpura area of the patient, and the organ function returned to normal. However, the patient was still in a coma after stopping sedative drugs. On the 7th day, head CT showed left cerebral hemisphere infarction with hemorrhage. The patient’s family discontinued treatment, and the patient was discharged. On follow-up, the patient unfortunately passed away on the day of discharge.

**Table 1:** Overview of crucial laboratory results.

	PLT	Lac	proBNP	Bil	ALT	AST	PT	APTT	Fib	d-d	PC	AT-III	EF	CI	SVRI
	*109/L	mmol/L	pg/mL	μmol/L	IU/L	IU/L	s	s	g/L	μg/mL	%	%	%	l/min/m^2^	dyne*s/cm^5^*m^2^
Case 1	13	10	>35,000	91	637	3115	31	59	8.3	>20	30	44	31	3.05	1114
Case 2	3	8.6	>35,000	108	62	104	18	70	3.2	>20	33	40	34	3.06	1354
Case 3	0.8	8	>35,000	98	54	46	18.1	48	2.7	>20	/	/	25	2.54	2179

ALT = alanine aminotransferase, APTT = activated partial thromboplastin time, AST = aspartate aminotransferase, AT- III = antithrombin III activity, Bil = bilirubin, CI = cardiac index, d-d = D-dimer, EF = ejection fraction, Fib = fibrinogen, Lac = lactic acid, PC = protein C activity, PLT = platelet count, proBNP = pro-brain natriuretic peptide, PT = prothrombin time, SVRI = systemic vascular resistance index.

**Table 2:** Published cases of SPG caused by urosepsis.

case	Author, year	Age/sex	Underlying disease	Pathogen	Method	DIC	Vasopressors (dose/duration)	Liver dysfunction	Cardiac dysfunction	AKI	Time Gap (BP↓ /VP to SPG)	Site	Amputation (Site/Time of amputation)	Outcome/Duration of survival
1	Mark et al, 1984^[4]^	83/M	N	*E coli*	Urine, blood culture	P	DA*1 d	N	Rapid atrial fibrillation	P	2	Fingers, toes (bilateral)	None	Death (day 56)
2	Leaker et al, 1987^[5]^	32/F	Spina bifida, ureteroileostomy, and recurrent urinary tract infection.	*Proteus mirabilis*	Blood and urine cultures	P	DA	NM	NM	P	1	Nose, trunk, bilateral hands and legs	P (both legs below the knee, right hand, and all digits of the left hand).	Survived
3	Mellor et al, 1999^[6]^	72/M	Renal calculus	*E coli*	Blood cultures	NM		NM	NM	P	4	Bilateral lower legs, feet, fingers	P (bilateral below-knee and digital/3week)	Survived
4	Ghosh et al, 2010^[7]^	15/F	NM	*Pseudomona*	Blood cultures	P	NM	NM	NM	NM	NM	Bilateral fingers and toes, nose	N	Survived
5	Helviz et al, 2011^[8]^	44/F	Renal calculus, urethral stents	*E coli*	Urine/blood cultures	p	NE, Vasopressin	NM	Hypokinesis	NM	1	Bilateral feet, fingers	P (left, below the knee/65 d, right metatarsal/86 d)	Survived
6	Markelov et al, 2012^[9]^	70/F	Congenital paralysis below the waste	*E coli*	NM	p	NE max 30 mcg/kg/min)vasopressin	NM	NM	NM	NM	Bilateral fingers and right toes	P (bilateral fingers and right toes)	Survived
7	Reid et al, 2013^[10]^	87/F	NM	*Proteus mirabilus*	Blood culture	P	NM	NM	Tachycardia	P	2d	The fingers and the toes (bilateral)	P (all gangrenous fingers)	Survived
8	Akamatsu et al, 2013^[11]^	50/M	NM	*E coli*	Blood culture	P	NEe, DA, and dobutamine*3d	NM	Hypokinesis	NM	3	Fingers, toes (bilateral)	P(all gangrenous fingers and toes)	Survived
9	Shimbo et al, 2015^[12]^	63/F	NM	*E coli*	Blood culture	P	Vasopressor therapy	NM	NM	NM	4	Fingers, toes (bilateral)	P (10 gangrenous areas on the peripheral extremities, and the left thumb was covered with a flap/day 36)	Survived
10	Phan et al, 2015^[13]^	35/F	An astrogliomand Crohn’s disease	N	Culture of all specimens	P	Noradrenaline	NM	NM	P	1	Forearms, hands, feet (bilateral)	P (debridement and amputation of selected digits of all 4 limbs)	Survived
11	Dong et al, 2015^[14]^	60/F	Multiple injuries	N	Culture of all specimens	P	DA4.8-29.5lg/kg/min*8d	NM	Tachycardia	NM	4	The fingers and the toes (bilateral)	P (all gangrenous fingers and toes/3 months)	Survived
12	Shin et al, 2015^[15]^	68/F	NM	NM	NM	NM	NE 3.49 mcg/kg/min*2d	NM	NM	NM	NM	Upper lip and all fingers and toes	NM	Survived
13	Jiang et al, 2017^[16]^	76/F	DM, hypertension, cardiac disease	*E coli*	Urine, blood culture	P	DA20 μg/kg/min, NE −.41 to 1.0 3μg/kg/min*1 wk	P	Rapid atrial fibrillation	P	2	Fingers, toes (bilateral)	P	Survived
14	Pankaj et al, 2018^[17]^	35/F	N	*E coli*	Urine culture	N	High dose of DA and NE*5 d	NM	N	N	1	Forearms, hands, feet (bilateral)	P (left below-elbow and right below-knee, all fingers and toes of the other 2 limbs/Within 25 days)	Survived
15	Jung et al, 2018^[18]^	46/F	NM	NM	NM	NM	NE32 mg*24 h	NM	Tachycardia	NM	8	Hands, wrists, lower legs, feet (bilateral)	P (bilateral carpal and knee joints below/after 4 wk)	Survived
16	Ruffin et al, 2018^[19]^	75/M	Hypertension, hyperlipidemia, nephrolithiasis	N	Culture of all specimens	NM	DA 2 µg/kg/min (max 20 µg/kg/min) and NE 1 µg/min (max 30 µg/min), vasopressin 2.4 units/h*2 d	NM	NM	NM	few days	Left hand, bilateral feet	P (all 3 digits of the left hand/5 mo, bilateral transmetatarsa/1 yr).	Survived
17	Ghosh et al, 2020^[20]^	49/F	NM	*Pseudomonas*	Blood cultures	P	NM	NM	NM	NM	NM	Leg, feet (bilateral)	NM	Death (day 4)
18	Hernandez et al, 2021^[21]^	42/F	Hypertension	*E coli*	Blood culture	P	NE*48 h	P	Tachycardia	P	1	Upper, lower extremities(bilateral)	P(the left hand partial amputation and debridement of 2 fingers of the right hand/A few months later)	Survived
19	Fukuta et al, 2021^[22]^	73/M	Chronic subdural hematoma, symptomatic epilepsy, and DM.	*Citrobacter freundii*	Urine cultures	P	NE, vasopressin*72 h	N	Tachycardia	P	4	Lower limbs (bilateral)	N	Death (day 25)
20	Yao et al, 2021^[23]^	48/F	Hypertension, diabetes	*Klebsiella pneumoniae*	Blood cultures	P	NE, DA	P	Tachycardia	P	2	Trunk, limbs	N	Death (day 4)
21	Lowry et al, 2022^[24]^	55/F	Hyperlipidemia, gastroesophageal reflux disease, and an anemia	*E coli*	Blood and urine cultures	P	NE*3d	P	Tachycardia	N	2	Left hand, right fingers, bilateral toes	P (five fingers on the left hand/day 24)	Survived
22	Lin et al, 2023^[25]^	59/F	Renal calculus	*E coli*	Blood cultures	P	NE1.6 µg/kg/min; epinephrine	P	Hypokinesis	P	3	Forearms, hand, bilateral feet, lower legs	P (below the knee, below the elbow/50d)	Survived
23	Our case 1	53/F	Renal calculus	*E coli*	Urine/blood cultures	P	NE max 2µg/kg/min, Metaraminol max 10µg/kg/min*3d	P	Hypokinesis	P	3	Bilateral hands and feet, trunk	N	Death (day 6)
24	Our case 2	59/F	Hypertension, brain stem hemorrhage	*E coli*	Blood cultures	P	Metanolamine max 8.33 µg/kg/min, NE max 1.67 µg/kg/min*5d	P	Hypokinesis	P	1	Bilateral fingers and toes	N	Death (day 5)
25	Our case 3	71/F	Hypertension, diabetes	*Klebsiella pneumoniae*	Blood cultures	P	NE maximum 0.11µg/kg/min, Metaraminol maximum 1.1 µg/kg/min*4 d	P	Hypokinesis	N	3	Fingers, toes (bilateral)	N (auto-amputation)	Survived

AKI = acute kidney injury, d = day, DA = dopamine, DIC = disseminated intravascular coagulation, DM = diabetes mellitus, *E coli* = *Escherichia coli*, F = female, h = hour, M = Male, max = maximum, N = negative, NE = norepinephrine, NM = Not mentioned, P = positive, time gap ↓BP/ VP to SPG = time interval (approximate) from hypotension (with or without start of vasopressors) to evidence of critical limb ischemia indicated symmetrical peripheral gangrene.

### 2.2. Case 2

A 59-year-old female was admitted to the intensive care unit with a 1-day history of fever and a 10-hour history of disturbance of consciousness. She had a history of hypertension and brainstem hemorrhage. The initial diagnosis was urosepsis and septic shock.

After admission, the blood, urine and sputum cultures were completed. Therapeutically, empirical anti-infection (meropenem), hydrocortisone, invasive ventilation, fluid resuscitation, step-up drugs (methanolamine and norepinephrine were titrated up to 8.33 µg/kg/min and 1.67 µg/kg/min, respectively), hydrocortisone, CRRT, plasma exchange and blood product infusion were used. At the same time, double-J catheters were placed bilaterally by urologists, and a large amount of milky white viscous liquid was drained out. On the same day, the patient had diffuse cyanosis in both hands and feet. The peripheral blood smear and Doppler ultrasonography of the extremities were normal. On day 3, the dose of vasopressor drugs was reduced but the test results remained unsatisfactory, some of the examination results are shown in Tables 1 and 2. The results of the blood and urine cultures suggested *Escherichia coli*. On the 5th day, the dose of vasopressor drugs was gradually reduced, norepinephrine was discontinued, and mehydroxylamine was maintained at 1.1 µg/kg/min. The organ function improved, and the cyanosis area was gradually limited to the tip of the fingertips. However, on the 6th day, the patient’s family gave up treatment due to financial difficulties, and the patient died on the 2nd day after discharge.

### 2.3. Case 3

A 71-year-old female was admitted to the intensive care unit with a 2-day history of vomiting and flank pain. She had a history of hypertension and diabetes. Extracorporeal lithotripsy of both kidneys was performed 2 weeks prior. The initial diagnosis was urosepsis and septic shock. After admission, we obtained cultures, initial empiric antimicrobial drugs (meropenem), hydrocortisone, fluid resuscitation, invasive ventilation, vasopressor drugs (Norepinephrine and Metaraminol were titrated to a maximum of 0.11 µg/kg/min, 1.1 µg/kg/min, respectively), optimized myocardial contractility, blood product infusion and CRRT were used. And the urologists placed a double-J stent bilaterally, and a large amount of milky viscous liquid was drained. By day 3, the second, third, fourth and fifth digits of her left hand and the third and fourth digits of her right hand exhibited progressive pallor. Over the next 2 days, the patient’s vasopressors were tapered and discontinued. However, the test results remained unsatisfactory, some of the examination results are shown in Tables 1 and 2. The peripheral blood smear and Doppler ultrasonography of the extremities were normal. The results of the blood culture suggested *Klebsiella pneumoniae*. On day 5, the patient’s overall organ function returned to normal and the skin at the end of her fingers had darkened and stretched upward, we administered heparin, B vitamins, papaverine, and peripheral heat preservation treatment. Over the next 3 weeks, the patient’s area of ischemia that was already ischemic progressed to gangrene with well-defined boundaries of the surrounding tissue but she refused to undergo an amputation. At 20 months of follow-up, the dry gangrene area of the patient’s right hand returned to normal. The dry gangrene area of the left hand with 4 fingers gradually self-amputated exposing the fifth phalanx. The patient self-amputated the fifth phalanx without any complication.

## 3. Discussion

To our knowledge, this is the first retrospective study and pooled analysis focusing on patients with urosepsis complicated with SPG to date. A review of the Chinese and English language literature was performed in the PubMed, Medline and WanFang databases using the keywords “Symmetric Peripheral Gangrene” or “Purpura Fulminans” and “sepsis” or “infect.” At the same time, we also reviewed the relevant articles in the references in the literature. The final search was performed on October 20, 2023. All statistical analyses were performed using the SPSS 26.0 software package (SPSS, Chicago, IL, USA), continuous variables were expressed as the means ± standard deviation.

Finally, this study included a total of 25 patients with SPG resulting from urosepsis, comprising 22 cases reported in other literature sources (n = 22 cases) and our present study (n = 3 cases) (Table 2). The following information was collected for each study: demographic data, cutaneous features, etiology, clinical features, treatments, prognosis.

### 3.1. Demographic data

Patients from the Asian region accounted for 16 cases, while the American and European regions reported 5 and 4 cases, respectively. The gender distribution was 5 males and 20 females, and the age at presentation was 56.80 ± 17.52 years. There were 6 patients with hypertension and 4 patients with diabetes mellitus in their past history. Our results revealed that the mean age of urosepsis patients with SPG was 56 years, and it tended to occur in adult patients of any age. There were far more female patients than male patients (male:female, 1:4), which may be related to the higher incidence of urinary tract infections in female patients,^[26]^ and Ghosh et al^[20]^ also found a female preponderance in their study.

### 3.2. Cutaneous features

In these cases, the symmetric distal skin of the patient was mostly purple or white, followed by cyanosis and extreme gangrene. Only 1 patient had unilateral gangrene of the lower extremity.^[9]^ Three of them had skin necrosis on the trunk in addition to gangrene of the limbs and distal organs. The skin lesions of 11 patients only existed in the extreme ends of the body, such as the tips of the hands, toes, nose and lips. The remaining 14 patients exhibited cutaneous lesions that extended beyond the digits.

### 3.3. Etiology

The microbiological results of 23 cases showed that *Escherichia coli* was the most common, being involved in 13 cases (56.52%), followed by Klebsiella pneumoniae, Pseudomonas, Proteus mirabilus, and Citrobacter freundii. In previous studies, Neisseria meningitidis was the most common microbe causing purpura explosion, followed by Streptococcus pneumoniae.^[3]^ However, our results suggest that patients with urosepsis combined with SPG have gram-negative bacterial infections, and *E coli* is the most common microbe.

### 3.4. Clinical features

According to the new diagnostic criteria for disseminated intravascular coagulation (DIC) from the Japanese Society on Thrombosis and Hemostasisor^[27]^ the diagnosis in the original case, only 1 case was not complicated with DIC. According to the 2012 guidelines for the diagnosis of abnormal liver function in sepsis^[28]^ or the diagnosis in the original case, the liver function indexes were available in 9 patients, suggesting that 7 patients had liver dysfunction, of which Lin et al^[25]^ and the 2 patients in this paper mentioned above had decreased PC activity and AT-III activity. In terms of hemodynamics, all known patients were treated with vasopressor therapy, and invasive hemodynamic monitoring showed normal or even low systemic peripheral vascular resistance (SVRI) and a low cardiac output index (CI) in our patients who were using vasopressors. Seven patients are known to have started to develop gangrene in the peripheral skin only after the dose of vasopressor was reduced or even discontinued.^[4,11,16,19,22]^ The findings from the summary of 20 cases of SPG caused by urosepsis showed an interval of 2.60 ± 1.70 days from the onset of hypotension/vasopressor therapy to the onset of ischemic limb necrosis. Among the patients in whom cardiac function was mentioned, 6 patients had diffuse cardiac hypokinesia^[8,11,25]^ and 2 patients had rapid atrial fibrillation.^[4,16]^

### 3.5. Pathogenesis

Markelov et al reported that gangrene occurred in other limbs of the patient, but gangrene was not found in the left lower limb due to chronic occlusion of the left external iliac artery.^[9]^ Does it suggest that the patient may have a low distribution of endotoxin and other factors in the right lower limb due to poor blood flow, thus allowing the patient to avoid gangrene? This is because severe infection can cause transient perivascular inflammation and increased vascular permeability, leading to vascular thrombosis. Our results showed that 6 patients had cardiac diffuse hypokinesia, and 2 patients had atrial fibrillation. This suggests that septic cardiomyopathy is an important factor that induces SPG, which may be due to elevated venous pressure in the case of severe right heart failure. Blood supply redistribution leads to reflexive collapse of the limb veins.^[29]^ In our study, 95% of patients had DIC and the fibrinogen level in many patients was usually within the normal range or even elevated because fibrinogen is an acute phase reactant and can be highly elevated in a proinflammatory environment. Hyperfibrinemia is an important risk factor leading to microthrombosis and vascular occlusion, which is also a factor that complicates SPG.^[30]^ Therefore, DIC is a key factor of SPG. Theodore E et al predicted that severe depletion of protein C and protein S would contributed to microvascular thrombosis. The half-lives of protein C and antithrombin III are 58 and 8 hours, respectively, so SPG episodes are usually observed for at least 2 days after shock and DIC in sepsis patients, and this is called “delayed”.^[31]^ In our study, it was concluded that SPG occurred 2.60 ± 1.70 days after shock (median), consistent with the “delayed” of SPG. It has been reported that AT-III levels and protein C levels in patients with sepsis can be reduced to 87% and 80% of those in healthy patients and to 60% of those in patients with severe sepsis.^[32]^ However, not all patients with sepsis have SPG. Consumption of natural anticoagulants is not the only factor contributing to SPG. Four of the patients discussed here had a history of diabetes mellitus, a well-established risk factor for the development of peripheral artery disease.^[33,34]^ The pathogenesis of peripheral artery disease in diabetic patients includes difficulty in controlling infection and promoting vascular inflammation and endothelial dysfunction, leading to vascular wall disorders.^[35]^ However, the proportion of patients with diabetes is not high; therefore, diabetes is not an important risk factor for SPG. At the same time, this study found that 11 patients underwent invasive urological surgery. Invasive urologic surgery is an effective method for the treatment of urinary calculi and hydronephrosis, but approximately 7.4% of patients with invasive urologic surgery will develop infection and sepsis.^[36]^ The incidence of sepsis is higher if surgical intervention is performed in the presence of active infection.^[37]^ Invasive urological surgery can aggravate sepsis and further aggravate the process of SPG. To summarize, the pathogenesis of urosepsis-induced SPG include the following: DIC, severe infection, natural anticoagulant depletion, hypoperfusion, cardiac dysfunction, cold, diabetes, urologic surgery, CRRT, etc.

### 3.6. Treatments

In addition to basic treatment, such as anti-infection, fluid resuscitation, transfusion of blood products and organ function support, 6 patients received heparin treatment, 11 patients underwent invasive urological surgery operation, 8 patients received renal replacement therapy, and 3 patients received plasma exchange. For DIC, if there is bleeding, replenishment of depleted clotting factors, such as intravenous plasma infusion, platelets, and plasma exchange, should be given. In cases of major thrombosis, several anticoagulants are available. Heparin may help stop the progression of gangrene. The DIC guidelines recommend the use of heparin in cases of predominantly thrombotic DIC,^[38]^ and the review of ischemic limb gangrene also supports the use of heparin.^[39]^ Importantly, heparin therapy is not contraindicated in patients with prolonged clotting tests because these disorders are a sign of blood clots, not a tendency to bleed.^[40]^ In addition, the effects of heparin may not be as good when there is inflammation^[41]^ and anti-Xa assays can be used to assess heparin levels.^[40]^ Ischemic limb injuries can occur rapidly due to a severe disorder of the procoagulant-anticoagulant system, even though it may be too late to start anticoagulation at the first sign of ischemic limb injury. The results of the PROWESS SHOCK test showed that drotrecogin alfa had no benefit in patients with sepsis.^[42]^ Subsequently, recombinant human activated protein C was withdrawn from the market, but the trial included patients with sepsis, not just patients with SPG secondary to sepsis. Meaghan et al summarized the research on PC concentrate in the treatment of purpura purpura and found that “treatment with PC concentrate can normalize PC levels and markers of DIC and possibly reduce morbidity and mortality.^[40]^” However, the small sample sizes and weak designs of these studies require further research.

Antithrombin (AT) and recombinant human soluble thrombomodulin are recommended for DIC patients with organ failure.^[43,44]^ Other treatments, such as sympathetic block,^[45]^ vasodilators, alpha receptor blockers,^[7]^ hyperbaric oxygen,^[46]^ paparine dextran and other measures,^[47,48]^ however, have not been proven effective. Early amputation should be avoided, as the extent of gangrene may not be as extensive as expected, allowing the formation of gangrene boundaries and automatic amputation.^[47]^ Early physical therapy can restore the mobility and range of motion of the joint.^[49]^

### 3.7. Follow-up and survival analysis

For patients with SPG caused by urosepsis, the survival rate was 76%. Amputation was needed in 94.44% of the surviving patients with limb gangrene. The median interval between limb ischemia and surgical amputation was 86 days. One patient had an automatic limb amputation, and the time period from diagnosis to the automatic limb detachment was approximately 20 months. Only 1 patient was complicated with osteomyelitis.^[21]^

## 4. Conclusion

Timely and accurate diagnosis, effective infection control, correction of hypoperfusion, organ function support, early management of DIC, avoidance of premature amputation and multidisciplinary comprehensive treatment are crucial for the successful treatment of SPG caused by urosepsis.

## Author contributions

**Data curation:** Xiujuan Xu, Gaofei Wu.

**Formal analysis:** Lianghua Zhu, Junjing Zha.

**Writing – original draft:** Yuanyuan Chen.

**Writing – review & editing:** Chuji Cheng, Kai Liu.
